# Physical Demands of Handball Referees: A Big Data Analysis between the EHF Men's and Women's EUROs 2022

**DOI:** 10.5114/jhk/202371

**Published:** 2025-10-01

**Authors:** Demetrio Lozano, Antonio Cartón-Llorente, Virgilio Gilart-Iglesias, Diego Marcos-Jorquera, Carmen Manchado

**Affiliations:** 1 Department of Health Sciences, San Jorge University, Zaragoza, Spain.; 2 Department of Computer Science and Technology, University of Alicante, San Vicente del Raspeig, Spain.; 3 Faculty of Education, University of Alicante, San Vicente del Raspeig, Spain.; 4 Methods Commission, European Handball Federation.

**Keywords:** time-motion analysis, worst-case scenario, physical fitness, running pace, refereeing

## Abstract

Handball referees face significant physical demands during the games that can influence the accuracy of their decisions. The aim of this study was to compare the movement profile of the referees during the men’s and women’s EHF 2022 EUROs. A total of 60 referees were evaluated using a local positioning system which provided data on their total distance, pace, accelerations, decelerations, and time spent in different speed zones. To automate the proposed methodology, a system was designed based on three phases: 1) capture of information on the activities and the context of the match through sensor networks, the LPS system and WebScraping techniques; 2) information processing based on Big Data Analytics; 3) extraction of results based on a descriptive analytics approach. The analysis revealed that referees in the women's tournament covered greater total and high-speed distances, but the accelerations were greater in the men's championship. Despite these differences, the referees’ running pace, analyzed in fixed 5-min time intervals, followed a similar pattern across tournaments, identifying the first 10 min of play as the most demanding scenarios. These findings highlight the importance of adapting physical training of referees according to the specific demands of each competition.

## Introduction

Handball is a dynamic and complex team sport played all over the world, from the amateur to the top level. In this sport, the importance of the referee is fundamental in ensuring fairness, safety and tactical development of the game. One of the most important roles of referees in elite handball is to enforce the rules of the game in an impartial and consistent manner (Philippe et al., 2009; Warner et al., 2013). Referees must be prepared to assess complex situations within seconds and make accurate decisions throughout the match.

In most team sports, referees are exposed to significant physical demands during competition, as they have to closely follow the movements of players and the ball (Sabag et al., 2023). Inadequate management of physical demands can have a negative impact on cognitive functions, which are essential for correct refereeing decisions (Belcic et al., 2020; Bloß et al., 2022; Fernandes da Silva et al., 2010). While physical fitness alone does not ensure superior refereeing, it plays a crucial role in closely following the game, especially during the most demanding phases. Moreover, higher levels of physical and mental stress have shown to significantly impact referees' accuracy and the error rate (Castillo-Rodríguez et al., 2023; Fernandes da Silva et al., 2010; Morillo et al., 2017).

Of note, the European Handball Federation (EHF) requires continental referees to pass a fitness test (i.e., 20-m shuttle-running test) to participate in its competitions (Leger et al., 1988). This underscores the importance of adequate aerobic fitness, specifying the last speed surpassed of 8.5 km/h for women and 9.5 km/h for men (eurohandball.com). In this regard, physical fitness of handball referees has been previously reported, showing an average fat-free mass index of 19.9 ± 2.6 kg•m^2^, the maximum HR of 187.2 ± 11.1 BPM (which was 98.1 ± 4.6% of their calculated maximum HR), maximum oxygen uptake (V̇O_2max_) of 44.6 ± 6.1 ml/kg/min, and a peak lactate of 9.2 ± 3.2 mmol•l^−1^ (Babity et al., 2022).

Although physiological match demands that a handball referee must overcome seem to be primarily of moderate intensity (96.4% of total match time below HR2mmol), there exists match phases of heavy (from HR2mmol until HR4mmol) and severe (above HR4mmol) intensity (2.3 and 1.3%, respectively) that should not be overlooked. Consequently, an extensive aerobic training regime, averaging 4.3 ± 2.0 hours per week, has been deemed necessary for referees at the continental and international levels (Babity et al., 2022). Furthermore, a specific warm-up protocol has been recently introduced to adequately prepare the musculoskeletal system of EHF referees for enduring the clusters of high-intensity actions that occur during a handball match (available at: https://s7da33d36d31fa01e.jimcontent.com/download/version/1691349406/module/18311555796/name/Referee%20Warm%20Up%20Program.pdf; accessed on: 14 December 2024), stressing the growing interest of international handball federations in the physical preparation of their referees as a key factor for injury prevention. However, few studies to date have analysed the most demanding phases (i.e., worst-case scenarios) in handball, and all of them focused exclusively on the physical demands of players (Carton-Llorente et al., 2023; García et al., 2022).

The integration of Local Positioning System (LPS) technology by the EHF, in collaboration with the Select® (Select Sport 1947: Glostrup, Denmark) and Kinexon®'s tracking system (Kinexon, Munich, Germany), has revolutionized handball analytics. This system enables the acquisition of vast volumes of real-time, raw kinematic data for both players and referees, significantly enhancing game analysis and performance evaluation. This technology reports accurate and valid data (Hoppe et al., 2018; Serpiello et al., 2018) and has been used in research on the physical demands of handball at different performance levels (Carton-Llorente et al., 2023; Font et al., 2021; García-Sánchez et al., 2023). However, the proposed analyses require a large amount of information to be processed from raw and heterogeneous data sources and formats. Therefore, it is necessary to propose a system that homogenizes and automates this process in order to obtain the information in a limited time and with adequate quality. As a result, a modular and integral architecture based on big data analytics has been designed. This architecture allowed us to analyse the physical demands of handball referees by measuring total distance covered, the pace (m•min^−1^), time at different running speeds, and the number of accelerations (both positive and negative) during matches in two recent elite championships.

This study aimed to compare the physical demands on handball referees in high-level male and female competitions and quantify the locomotion worst-case scenarios in fixed 5-min windows for both tournaments. It was also hypothesized that the running pace of referees during high-level matches would follow an identifiable pattern. A better understanding of these demands would help coaches and physical preparation managers to design specific training programs for referees that optimize performance, improve their decision-making, and minimize the risk of injury.

## Methods

### Participants

**Figure 1:**
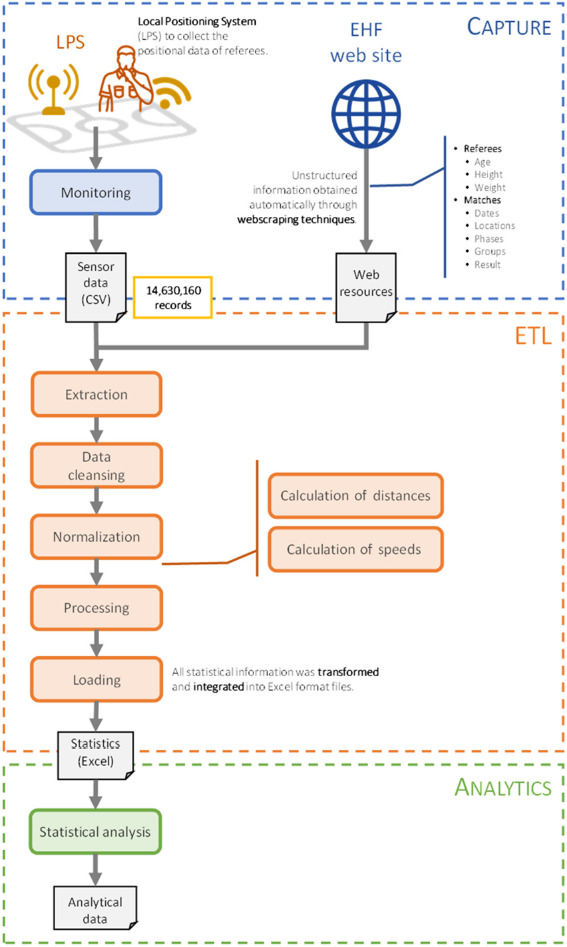
Methodology of the comprehensive handball system proposed.

During 112 matches of the EHF Men's 2022 EURO and EHF Women's 2022 EURO, 60 referees were assessed using a local positioning system (®KINEXON) affixed to their upper body. Table 1 outlines the anthropometric characteristics and age of the referees. The official statistical records supplied by the EHF and routine monitoring of referees during the competition (Figure 1, Data Capture Layer) provided all data (14,630,160 records). This study was conducted following the principles of the Declaration of Helsinki and approved by the Institutional Review Board of the University of Alicante, San Vicente del Raspeig, Spain (protocol code UA-2020-09-10, approval date: 2 November 2020). Informed consent was obtained from all participants included in the study.

**Table 1:** Referees’ anthropometric characteristics (mean ± standard deviation).

Variable	EHF Men’s EURO 2022 n = 36 (m: 34; f:2)	EHF Women’s EURO 2022 n = 24 (m: 8; f:16)	*p*-value
Age (years)	40.4 ± 4.6	37.6 ± 2.6	0.065
Body Height (cm)	183.3 ± 7.2	174.5 ± 7.3	<0.001*
Body Mass (kg)	87.6 ± 11.1	70.1 ± 10.5	<0.001*
BMI (kg•m^2^)	26.0 ± 2.6	22.9 ± 2.1	0.004*

* Statistical differences between groups; m: male referees; f: female referees

### Measures

Each competition match tracking of the 3D position data (x / y / z) of each referee was obtained in real time using a portable inertial sensor (Kinexon SafeTag, Kinexon Precision Technologies, Munich, Germany) that was included in a top worn under the shirt and fitted between the shoulder blades to avoid discomfort to the referees. This device provides 9-axis inertial data (accelerometer, gyroscope, magnetometer) capable of recording accelerations/decelerations, rotation, and yaw angles (roll, pitch, yaw) with a refresh rate of up to 60 Hz. This instrument has an adequate inter-device reliability (coefficient of variation around 5%) for the analysis of physical demands in team sports (Alt et al., 2020; Hoppe et al., 2018; Manchado et al., 2021).

The following performance metrics were measured for each referee during each match according to the official EHF timekeeping: total distance covered (m), pace (m•min^−1^), accelerations (n), decelerations (n), changes of direction (n) and time (s) spent in the following speed zones: standing (<0.9 m•s^−1^), walking (1.0–1.9 m•s^−1^), jogging (2.0–3.9 m•s^−1^), running (>4.0 m•s^−1^). The pace was calculated as the ratio between distance covered and playing time, and the running time category also encompassed high-intensity running (5.5–6.9 m•s^−1^) and sprinting (>7 m•s^−1^), since such high-intensity runs, although they do differ in previous studies on player demands (Bělka et al., 2016; Cardinale et al., 2017), hardly occurred among the referees. Following the same criteria, all acceleration changes greater than 2 m•s^−1^ (positive and negative) were considered in a single category (referred to as "accelerations" from now on) and changes in direction recorded were also added to represent in a single value all high intensity actions.

The Kinexon system operates by means of triangulations between nine antennae located around the handball court and connected to a server, and ten reference antennae acting as anchors. Setting, calibration and verification of the LPS system in all championship facilities followed the procedure described in the study by Manchado et al. (2021).

The rest of the data necessary to perform the physical demand analysis was collected from the EHF website using WebScraping techniques.

### Design and Procedures

In order to obtain and compare the physical demands of handball referees during male and female high-level competition an architecture based on Sensors Network, LPS and Big Data Analytics was designed following the methodology described in Figure 1.

For data processing, the collected data were cleaned up and normalized before proceeding to the processing phase (Figure 1).

Finally, in order to perform the subsequent data analysis, we conducted the loading process and all the information necessary for the study was transformed into Excel format files using an input format compatible with the statistical analysis tool.

Firmware and software versions employed in this study corresponded to the latest update of the aforementioned company (2019).

### Statistical Analysis

Descriptive characteristics are presented as means and standard deviations (SD). The Student's *t*-test was conducted to evaluate a priori differences between the referees in the male and female tournaments. The Kolmogorov-Smirnov test was performed to confirm data distribution normality and the Mann-Whitney U test was applied to evaluate between-group differences for all studied variables. Therefore, the subsequent descriptive analysis was presented as medians (Mdn) and interquartile ranges (IQR). Effect sizes for all pairwise comparisons were calculated using U and Z values to estimate biserial correlation (r). To gauge the strength of each relationship, we employed a revised categorization for effect size (negligible <0.2, small 0.21–0.6, moderate 0.61–1.2, substantial 1.21–1.99, and exceedingly substantial >2.0) as proposed within the field of sports sciences (Hopkins et al., 2009). Finally, mean and peak demands for the analysed variables were averaged for each of the 12 fixed 5-min time periods of a handball game. The segmentation was performed following the official playing time and only recordings lasting ≥ 270s (for each 300-s time window) were included in the final analyses.

## Results

**Figure 2:**
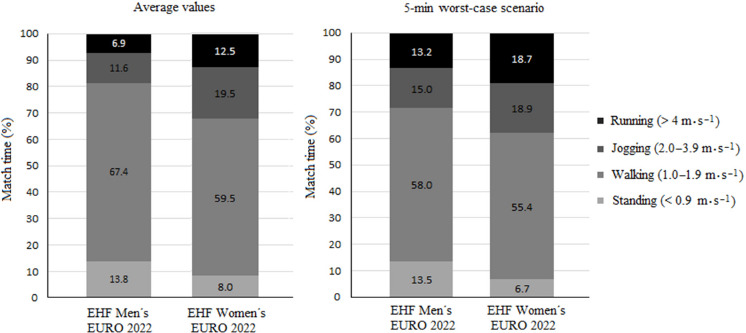
Time-motion analysis of handball referees during EHF Men’s and Women’s EUROs, divided into speed zones. Average values (left) and worst-case scenario values (right).

**Figure 3:**
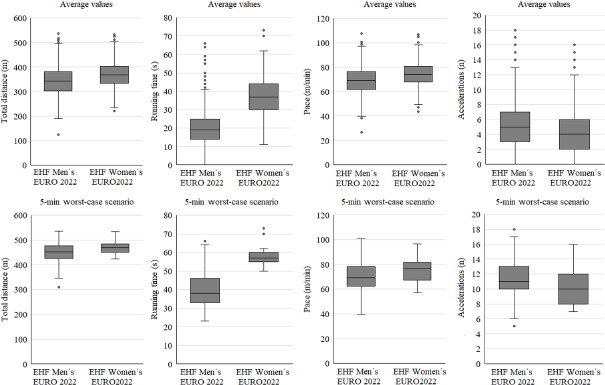
Physical demands of handball referees during EHF Men's and Women's EUROs 2022. Average values (upper line) and maximum values (lower line).

**Figure 4:**
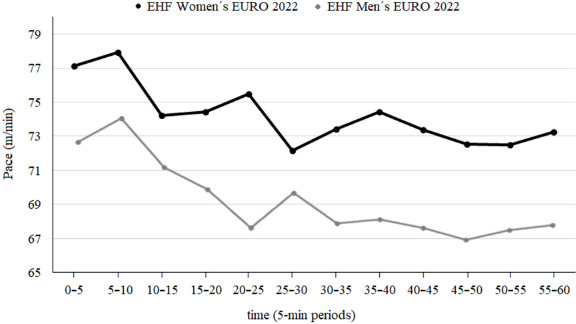
Physical evolution of the referee’s average pace during EHF Men's and Women's EUROs 2022.

The conducted analysis revealed that referees' time-motion demands were different between the EHF Men’s and Women’s EUROs 2022. Table 2 reports the differences in the referees’ physical demands during the male and female tournaments. The physical demands of referees by speed zones are shown in Figure 2, while the movement characteristics of referees in fixed 5-min windows, both in average and peak demands, are displayed in Figure 3. In addition, Table 3 reveals the differences (in %) between average and peak demands for the aforementioned 5-min fixed time spans. Finally, Figure 4 shows the evolution of the moving pace of referees over the 60 min of an international match, differentiating between the men's and women's EHF EURO 2022.

**Table 2:** Comparative analyses in time-motion variables between the EHF Men’s and Women’s EUROs 2022.

Variable	EHF Men’s EURO 2022 n = 36 Mdn (IQR)	EHF Women’s EURO 2022 n = 24 Mdn (IQR)	Difference (%)	U	*p*	r
Total distance (m)	4128 (697)	4471 (484)	8.3	650	0.001*	0.38 S
Standing time (s)	429 (546)	285 (161)	−33.6	285	0.023*	0.29 S
Walking time (s)	2587 (573)	2172 (211)	−19.0	217	0.001*	0.46 S
Jogging time (s)	367 (94)	691 (209)	88.3	844	<0.001**	0.77 M
Running time (s)	223 (62)	458 (78)	105.4	820	<0.001**	0.83 M
Pace (m•min)	68.7 (11.7)	74.1 (5.5)	7.9	606	0.009*	0.35 S
Accelerations (n)	67 (36)	61 (21)	−9.0	306	0.066	0.22 N

* p < 0.05, ** p < 0.001; Mdn: median; IQR: interquartile range; Standing (<0.9 m•s^−1^); Walking (1.0–1.9 m•s^−1^); Jogging (2.0–3.9 m•s^−1^); Running (> 4.0 m•s^−1^); S: small; M: moderate

**Table 3:** Worst-case scenario analysis of referees’ physical demands during EHF men’s and women’s EUROs 2022. Comparison between average and maximum 5-min demands.

	EHF Men’s EURO 2022			EHF Women’s EURO 2022		
Variable	5-min average Mdn (IQR)	5-min WCS Mdn (IQR)	Difference (%)	5-min average Mdn (IQR)	5-min WCS Mdn (IQR)	Difference (%)
Total distance (m)	344 (78)	452 (53)	31%	367 (70)	469 (34)	28%
Running time (s)	19 (11)	38 (13)	100%	37 (14)	57 (5)	54%
Pace (m•min)	68.7 (11.7)	69.3 (15.8)	1%	74.1 (5.5)	76.7 (14.6)	4%
Accelerations (n)	5 (4)	11 (3)	120%	4 (4)	10 (4)	150%

WCS: worst-case scenario, Running (> 4.0 m•s^−1^)

## Discussion

This research is the first to compare the physical demands of handball referees across two top level international men's and women's competitions. The main findings included significant differences that were found in the physical demands of referees between the male and female tournaments, with the referees in the female competition covering greater total and high-speed distances, and with effect sizes ranging from small to moderate (0.3–0.8). Additionally, analysis of worst-case scenarios in fixed 5-min intervals revealed that referees in both tournaments experienced increases of ~50% to 150% in high-intensity actions compared to the average demands for time intervals of the same duration. Finally, despite the differences found in physical demands between the men's and women's tournaments, the running pace of referees appeared to follow a similar pattern during the 60 min of gameplay, regardless of the competition.

### Physical Demands

Regarding the physical demands in competition, several studies have described the time-movement variables in referees (Babity et al., 2022; Belcic et al., 2020; Fernandes da Silva et al., 2010). Those studies described that the intensities of a handball competition were moderate for 96.4% of the total playing time, which coincides with our results in both male (standing 13.8%, walking 67.4%, jogging 11.6%) and female (standing 8%, walking 59.5%, jogging 19.5%) competition (Figure 2). This may be due to the intermittent characteristics of handball, which require players to undergo demanding physical confrontations followed by variable recovery periods (Fasold and Redlich, 2018), the relatively small playing area (40 x 20 m) and the involvement of two referees in the game.

The physiological demands of refereeing top-level handball matches have been previously categorized based on the HR, showing time spent at heavy and severe intensities was small (2.3% and 1.3%, respectively) (Fernandes da Silva et al., 2010). The results of the current work, based on the LPS tracking system, present higher values of time spent at high-intensity, representing 6.9% of total time in the EHF Men's EURO 2022 and 12.5% of total time in the EHF Women's EURO 2022. Despite the advantage of having real-time heart rate responses to exercise, the HR has shown certain limitations for workload quantification as a standalone method (Achten and Jeukendrup, 2003). Of note, the HR increases with some delay and does not adjust well to sudden changes in intensity (e.g., short-duration intermittent activities). In addition, the aforementioned study of Fernandes da Silva et al. (2010) displayed peak velocities up to 12.5 ± 1.0 km•h^−1^, whereas our velocity-based threshold for high-intensity activity was actually higher (14.4 km•h^−1^). Consequently, the findings provided in this research seem to represent the physical demands of continental-level handball referees more accurately.

It is worth noting that physical demands of male and female players in high level international championships have been previously studied (García-Sánchez et al., 2023; Gómez-López et al., 2024). When comparing them by gender, García-Sánchez et al. (2023) confirmed that the total distance covered was moderately greater in female (4549.1 ± 758.6 m) than in male competitions (3332.6 ± 1257.6 m), and that the running pace was largely higher in female (110.5 ± 7.2 m•min^-1^) compared to male matches (78.4 ± 19.7 m•min^−1^) (ES = 1.6). The results of that systematic review are consistent with our results in terms of the total distance covered by referees involved in male and female elite level matches, even though referees do not make substitutions and have to referee the entire match (60 minutes).

Although the distance covered by handball referees in each offence-defence phase is only 20 m, as they have to cover the distance from the end line to the midfield line, referees’ physical demands seem to mimic those of players involved in the game. In light of these results, the differences between the physical demands of the men's and women's championships must be taken into account in order to adjust the physical preparation of referees, as poorer physical ability could have an impact on decision making during the game (Belcic et al., 2020; Bloß et al., 2020).

#### Worst-Case Scenario

Regarding the most demanding intervals of the game, no research was found that analysed the worst-case scenario in handball refereeing. However, previous research on rugby (Igoe and Browne, 2020) and indoor soccer (Martinez-Torremocha et al., 2023) including worst-case scenario analysis in fixed 5-min windows showed similar demands for indoor soccer referees regarding the pace (95 m•min^−1^) (Martinez-Torremocha et al., 2023), but higher for rugby referees (107.9 m•min^−1^) (Igoe and Browne, 2020). These results might be explained by the dimensions of the field and the number of referees involved in each discipline.

Considering worst-case scenario analyses of handball players in high-level championships, we have found only two recent studies (Carton-Llorente et al., 2023; García et al., 2022) that describe the variables of each playing position. The results of those studies confirm that male players cover four times more distance at high intensity than referees in the same championship. Those results are therefore difficult to compare with ours. As it is stated, most of the efforts made by handball referees are of low intensity (Fernandes da Silva et al., 2010).

Of note, current results show a more demanding worst-case scenario in women's championships. These findings are consistent with greater high-intensity physical demands in the women's championships. This may be due to the higher pace of play (Fasold and Redlich, 2018). Compared to the men's Euro 2022 player data, where the median peak acceleration was 2.71 m•s^2^ [IQR (0.49)] and the median peak deceleration was 2.24 m•s^2^ [IQR (2.75)], similar data appeared in the women's Euro 2022, where the median peak acceleration was 2.67 m•s^2^ [IQR (0.52)] and the median peak deceleration was 2.14 m•s^2^ [IQR (2.85)]. Interestingly, the acceleration variable was higher in the men's championships. It is possible that these championships had less rhythm but higher accelerations due to the explosive and intermittent nature of handball. These findings are in line with those of Michalsik and Aagaard (2015) who observed significant gender differences in physical demands of elite team handball, with more high-intensity strength-related game actions and high-intensity running in the men's championships than in the women's championships. In contrast, women's championships involved a greater total distance covered and a greater relative workload than men's championships.

The results of this study should help improve the design of the physical preparation of referees in elite championships of different genders to meet their needs and to pay attention to injury prevention, as this can directly affect performance (Babity et al., 2022; Bloß et al., 2020; Heyn and Fleckenstein, 2021).

### Running Pace

Our results show differences in physical demands between the men's and women's tournaments, but the referees' running pace appears to follow a similar pattern throughout the 60 min of play, regardless of the competition. In the second periods the total distance per minute decreased in both championships. These results are in line with other studies (Belcic et al., 2020; Gutiérrez-Vargas et al., 2021), yet in order to understand them, more studies need to be carried out that jointly analyse the pace of play of players and referees.

In addition, monitoring physical demands of referees may reveal the true pace of matches, as they do not experience the continuous changes that players do.

A limitation of this study is the absence of physiological data. The inclusion of heart rate monitoring as a complementary method could enhance the complete understanding of physical demands of handball referees. Furthermore, the absence of control over external factors that could mitigate variability in playing conditions, such as the tournament phase, the level of competition, and the tournament type, implies that factors like the score of each 5-min set, the teams' playing style or the coaches' tactical decisions may have significantly influenced the referees' physical demands.

## Conclusions

In conclusion, our data describe the differences in physical demands on referees between men's and women's tournaments, with referees in the women's competition covering greater total distances and distances at high intensity. Worst-case scenarios at fixed 5-min intervals revealed that referees in both tournaments experienced increases of ~50% to 150% in high-intensity actions compared to average demands for time intervals of the same length. The referees' running pace appeared to follow a similar pattern over the 60 min of the match, regardless of the competition. These results have direct implications for the design and planning of specific training for referees in order to correctly support the physical demands of the pace of play, worst-case scenarios and acceleration-deceleration at specific moments of the competition. The information reported here addresses the growing concern of international handball federations to care for the physical condition of their referees, aiming to enhance their performance and keep them injury-free.

## References

[ref1] Achten, J., & Jeukendrup, A. E. (2003). Heart rate monitoring: applications and limitations. *Sports Medicine*, 33, 517–538. 12762827 10.2165/00007256-200333070-00004

[ref2] Alt, P. S., Baumgart, C., Ueberschär, O., Freiwald, J., & Hoppe, M. W. (2020). Validity of a local positioning system during outdoor and indoor conditions for team sports. *Sensors*, 20(20), 5733. 10.3390/s20205733 33050174 PMC7601858

[ref3] Babity, M., Zámodics, M., Lakatos, B. K., Rákóczi, R., König, A., Menyhárt-Hetényi, A., Fábián, A., Kiss, A., Tokodi, M., & Kovács, A. (2022). Cardiorespiratory fitness status of elite handball referees in Hungary. *Plos One*, 17(7), e0270999. 35797392 10.1371/journal.pone.0270999 PMC9262183

[ref4] Belcic, I., Ruzic, L., & Marosevic, A. (2020). Influence of functional abilities on the quality of refereeing in handball. *Baltic Journal of Health and Physical Activity*, 12(3), 3.

[ref5] Bělka, J., Hůlka, K., Šafář, M., Dušková, L., Weisser, R., & Riedel, V. (2016). Time-motion analysis and physiological responses of small-sided team handball games in youth male players: Influence of player number. *Acta Gymnica*, 46(4), 201–206.

[ref6] Bloß, N., Loffing, F., Schorer, J., & Büsch, D. (2022). Impact of psychological and physical load on the decision-making of top-class handball referees. *International Journal of Performance Analysis in Sport*, 22(3), 352–369. 10.1080/24748668.2022.2061323

[ref7] Bloß, N., Schorer, J., Loffing, F., & Büsch, D. (2020). Physical load and referees’ decision-making in sports games: A scoping review. *Journal of Sports Science & Medicine*, 19(1), 149–157. 32132838 PMC7039031

[ref8] Cardinale, M., Whiteley, R., Hosny, A. A., & Popovic, N. (2017). Activity profiles and positional differences of handball players during the World Championships in Qatar 2015. *International Journal of Sports Physiology and Performance*, 12(7), 908–915. 10.1123/ijspp.2016-0314 27918655

[ref9] Carton-Llorente, A., Lozano, D., Iglesias, V. G., Jorquera, D., & Manchado, C. (2023). Worst-case scenario analysis of physical demands in elite men handball players by playing position through big data analytics. *Biology of Sport*, 40(4), 1219–1227. 37867747 10.5114/biolsport.2023.126665 PMC10588589

[ref10] Castillo Rodríguez, A., Rodríguez-Caparrós, J. L., Figueiredo, A., González-Fernández, MD, F. T., Onetti-Onetti, W. (2023). Cause-Effect: The Relationship between Role and Experience with Psychological and Physical Responses in the Competition Context in Soccer Referees. *Journal of Human Kinetics*, 89, 289–300. 10.5114/jhk/169174 38053965 PMC10694706

[ref11] Fasold, F., & Redlich, D. (2018). Foul or no foul? Effects of permitted fouls on the defence performance in team handball. *Journal of Human Kinetics*, 63(1), 53–59. 10.2478/hukin-2018-0006 30279941 PMC6162971

[ref12] Fernandes da Silva, J., Castagna, C., Carminatti, L. J., Foza, V., Guglielmo, L. G. A., & de Oliveira, F. R. (2010). Physiological demands of team-handball referees during games. *Journal of Strength &and Conditioning Research*, 24(7), 1960–1962. 10.1519/JSC.0b013e3181ddb019 20555278

[ref13] Font, R., Karcher, C., Reche, X., Carmona, G., Tremps, V., & Irurtia, A. (2021). Monitoring external load in elite male handball players depending on playing positions. *Biology of Sport*, 38(3), 475–481. 10.5114/biolsport.2021.101123 34475629 PMC8329973

[ref14] García, F., Fernández, D., Illa, J., Reche, X., Font, R., Guitart, M., Pla, F., Tarragó, J. R., & Vázquez-Guerrero, J. (2022). Comparing the most demanding scenarios of official matches across five different professional team sports in the same club. *Apunts Sports Medicine*, 57(215), 100390. 10.1016/j.apunsm.2022.100390

[ref15] García-Sánchez, C., Navarro, R. M., Karcher, C., & de la Rubia, A. (2023). Physical demands during official competitions in elite handball: a systematic review. *International Journal of Environmental Research and Public He*alth, 20(4), 3353. 36834047 10.3390/ijerph20043353 PMC9965087

[ref16] Gómez-López, M., Rivilla-García, J., González-García, I., Sánchez-López, S., Angosto, S. (2024). Analysis of Spatial Offensive Performance in Handball: Differences between Men's and Women's Senior World Championships. *Journal of Human Kinetics*, 90, 169–182. 10.5114/jhk/170233 38380305 PMC10875690

[ref17] Gutiérrez-Vargas, R., Ugalde-Ramírez, J. A., Rojas-Valverde, D., Müller-Thyssen, M., & Pino-Ortega, J. (2021). External and internal load of Costa Rican handball referees according to sex and game periods. *E-Balonmano Com*, 17(2), 153–162.

[ref18] Heyn, J., & Fleckenstein, J. (2021). Incidence of injury and pain in referees in German national handball leagues: a cohort study. *BMC Sports Science, Medicine and Rehabilitation*, 13(1), 1–7. 10.1186/s13102-021-00320-1 PMC8359112 34384489

[ref19] Hopkins, W. G., Marshall, S. W., Batterham, A. M., & Hanin, J. (2009). Progressive statistics for studies in sports medicine and exercise science. *Medicine and Science in Sports and Exercise*, 41(1), 3–13. 10.1249/MSS.0b013e31818cb278 19092709

[ref20] Hoppe, M. W., Baumgart, C., Polglaze, T., & Freiwald, J. (2018). Validity and reliability of GPS and LPS for measuring distances covered and sprint mechanical properties in team sports. *PloS One*, 13(2), e0192708. 29420620 10.1371/journal.pone.0192708 PMC5805339

[ref21] Igoe, B. A., and Browne, D. (2020). Maximum ball in play demands for sub-elite Rugby Union referees in domestic club rugby. *Journal of Human Sport and Exercise*, 15(3), 478–488. 10.14198/jhse.2020.153.01

[ref22] Leger, L. A., Mercier, D., Gadoury, C., & Lambert, J. (1988). The multistage 20 metre shuttle run test for aerobic fitness. *Journal of Sports Science*s, 6(2), 93–101. 3184250 10.1080/02640418808729800

[ref23] Manchado, C., Pueo, B., Chirosa-Rios, L. J., & Tortosa-Martínez, J. (2021). Time–Motion Analysis by Playing Positions of Male Handball Players during the European Championship 2020. *International Journal of Environmental Research and Public Health*, 18(6), 2787. 10.3390/ijerph18062787 33801814 PMC8002104

[ref24] Martinez-Torremocha, G., Sanchez-Sanchez, J., Alonso-Callejo, A., Martin-Sanchez, M. L., Serrano, C., Gallardo, L., Garcia-Unanue, J., & Felipe, J. L. (2023). Physical Demands in the Worst-Case Scenarios of Elite Futsal Referees Using a Local Positioning System. *Sensors*, 23(21), 8662. 37960362 10.3390/s23218662 PMC10648636

[ref25] Michalsik, L. B., & Aagaard, P. (2015). Physical demands in elite team handball: Comparisons between male and female players. *Journal of Sports Medicine and Physical Fitness*, 55(9), 878–891. 24947813

[ref26] Morillo, J. P., Reigal, R. E., Hernández-Mendo, A., Montaña, A., & Morales-Sánchez, V. (2017). Decision-making by handball referees: design of an ad hoc observation instrument and polar coordinate analysis. *Frontiers in Psychology*, 8, 1842. 29104553 10.3389/fpsyg.2017.01842 PMC5655026

[ref27] Philippe, F. L., Vallerand, R. J., Andrianarisoa, J., & Brunel, P. (2009). Passion in referees: Examining their affective and cognitive experiences in sport situations. *Journal of Sport and Exercise Psychology*, 31(1), 77–96. 19325189 10.1123/jsep.31.1.77

[ref28] Sabag, E., Lidor, R., Arnon, M., Morgulev, E., Bar-Eli, M. (2023). Teamwork and Decision Making among Basketball Referees: The 3PO Principle, Refereeing Level, and Experience. *Journal of Human Kinetics*, 89, 313–326. 10.5114/jhk/169439 38053959 PMC10694708

[ref29] Serpiello, F. R., Hopkins, W. G., Barnes, S., Tavrou, J., Duthie, G. M., Aughey, R. J., and Ball, K. (2018). Validity of an ultra-wideband local positioning system to measure locomotion in indoor sports. *Journal of Sports Sciences*, 36(15), 1727–1733. 10.1080/02640414.2017.1411867 29192842

[ref30] Warner, S., Tingle, J. K., and Kellett, P. (2013). Officiating attrition: Considering the experiences of referees from a sport development perspective. *Journal of Sport Management*, 27(4), 316–328.

[ref31] Winter, E. M., and Maughan, R. J. (2009). Requirements for ethics approvals. *Journal of Sports Sciences*, 27(10), 985–996. 10.1080/02640410903178344. 19847681

